# Unveiling the efficacy of paroxetine and gabapentin in ulcerative colitis patients in remission with co-existing IBS-like symptoms: a single-blinded randomized clinical trial

**DOI:** 10.3389/fmed.2024.1468885

**Published:** 2024-11-20

**Authors:** Farahnaz Safaei, Shabnam Shahrokh, Nosratollah Naderi, Reyhaneh Rastegar, Alireza Shamsi

**Affiliations:** ^1^Gastroenterology and Liver Diseases Research Center, Research Institute for Gastroenterology and Liver Diseases, Shahid Beheshti University of Medical Sciences, Tehran, Iran; ^2^Department of Psychiatry, Taleghani Hospital, Faculty of Medicine, Shahid Beheshti University of Medical Sciences, Tehran, Iran

**Keywords:** gabapentin, irritable bowel disease, paroxetine, selective serotonin reuptake inhibitors, serotonin and noradrenaline reuptake inhibitors

## Abstract

**Introduction:**

This clinical trial is designed to explore the efficacy of paroxetine and gabapentin in alleviating functional gastrointestinal symptoms, anxiety, depression, and quality of life in patients with ulcerative colitis during the remission stage.

**Methods:**

The study enrolled 97 patients with ulcerative colitis in remission who had reported functional gastrointestinal symptoms. Patients were measured in terms of quality of life, anxiety, depression, and IBS severity. One group received paroxetine at a dosage of 20 mg/day, and the other group received gabapentin at a dosage of 100 mg/day in the first month and 300 mg/day in the second and third months. The patients were followed up for 3 months.

**Results:**

Anxiety (*p* < 0.001), depression (*p* = 0.012), and severity score levels (*p* = 0.045) among patients in the paroxetine group were significantly lower compared to the gabapentin group following the intervention. Paired evaluation in each treatment group revealed a significant reduction in the paroxetine group, while changes in the gabapentin group were not significant. Quality-of-life scores among patients in the paroxetine group were significantly higher compared to the gabapentin group following the intervention (*p* < 0.001).

**Conclusion:**

The rate of improvement in gastrointestinal functional symptoms, anxiety, depression, and quality of life is significantly superior with paroxetine compared to gabapentin.

**Clinical trial registration:**

https://irct.behdasht.gov.ir/trial/69397, identifier RCT20220417054557N1.

## Introduction

Ulcerative colitis (UC) is an inflammatory bowel disease characterized by continuous mucosal inflammation extending from the rectum to the proximal colon (1). UC is influenced by various factors, including genetics, environmental influences, changes in the intestinal microbiota, and the reactivity of the intestinal immune system (2). This disease follows a relapsing-remitting course (3). During inactive periods or remission, patients experience complete resolution of symptoms. Stool frequency returns to normal without bleeding or urgency, the colonic mucosa appears normal upon endoscopy, and the sigmoidoscopy score falls within the range of 0 or 1 according to the Mayo score (4).

One of the challenges in managing UC is the presence of functional gastrointestinal symptoms among patients during UC remission (5). Some UC patients in remission report frequent abdominal symptoms such as pain and discomfort along with changes in bowel habits, referred to as “irritable bowel syndrome (IBS)-like symptoms” (6). The prevalence of these symptoms has been reported to range from 9 to 46% (7). Investigations into their etiology suggest common factors shared between UC and IBS, including mucosal inflammation, alterations in the microbiome, increased intestinal permeability, genetic factors, and, importantly, interactions within the brain-gut axis (5). Several pathogenic mechanisms explain why some UC patients in remission experience symptoms of functional origin. These mechanisms include persistent inflammation, dysmotility, heightened visceral sensitivity, increased mucosal permeability following UC, microbiota disturbances, and psychological distress (8). Various interventions have been introduced to manage IBS-like symptoms in UC patients during remission. These interventions encompass psychological therapies, probiotics, fecal microbial transplantation, low fermentable oligosaccharides, disaccharides, monosaccharides and polyols (FODMAP) diets, and antidepressants. However, controlled clinical trials evaluating the effectiveness of these interventions in UC patients experiencing IBS-type symptoms are limited (5, 8, 9).

Psychological problems, such as anxiety and depression, plays a significant role in the development of functional gastrointestinal symptoms in both IBS and UC, influencing both their production and perception (5, 10, 11). Additionally, the presence of gut-brain axis dysfunction has been established in both IBS and IBD (12). As a result, antidepressants are considered a suitable treatment option due to their capacity for neuromodulation (13) and anti-inflammatory effects (14) and their positive impact on gut-brain axis disorders (15).

Selective serotonin reuptake inhibitors (SSRIs) and serotonin and noradrenaline reuptake inhibitors (SNRIs) represent two classes of antidepressants known for their effectiveness in alleviating both functional and anti-inflammatory symptoms in the digestive system. SSRIs and SNRIs act on the brain’s neurotransmitter systems to alleviate depressive symptoms. SSRIs primarily target the serotonin transporter, elevating serotonin levels, while SNRIs target both serotonin and noradrenaline transporters, increasing levels of both neurotransmitters. This distinction influences their efficacy and side effect profiles, with SSRIs being associated with lower seizure risk but higher rates of sexual dysfunction and weight gain, whereas SNRIs may be more effective for depression with anxiety symptoms and have a lower risk of sexual dysfunction but higher rates of nausea, dizziness, and dry mouth. Notably, their impact on nocturnal urinary frequency varies, with sertraline users experiencing increased frequency compared to duloxetine users (16–18). Paroxetine and gabapentin are both medications utilized in treating certain aspects of ulcerative colitis. Although differing in their primary neuroscientific aspects and clinical applications, they share some similarities in their potential therapeutic roles in managing specific aspects of ulcerative colitis. Paroxetine primarily targets the psychological and emotional dimensions of the condition by modulating serotonin levels in the brain, while gabapentin focuses on alleviating chronic pain and discomfort by modulating neurotransmitter activity in the central nervous system (19–22).

A comprehensive review of previous literature reveals a gap in understanding the comparative effectiveness of SSRIs and other medications like gabapentin in managing IBS-like symptoms in UC patients. While several studies have highlighted the potential benefits of SSRIs in improving gastrointestinal symptoms, less is known about the specific roles of paroxetine and gabapentin in this context, emphasizing the novelty of the present study. This study assessed the efficacy of paroxetine and gabapentin among UC patients in remission on the IBS-like symptoms, psychological factors, and overall quality of life. Paroxetine was selected for its effectiveness in modulating serotonin levels and its documented anti-inflammatory properties, which may contribute to alleviating symptoms in UC patients. Gabapentin, on the other hand, is known for its efficacy in managing neuropathic pain and discomfort associated with functional gastrointestinal symptoms, making it a suitable choice for this study (23–25).

## Patients and method

### Study design

The present study is a randomized clinical trial, single-blinded, with a parallel group. For blinding, the person who evaluated the outcome after 3 months was blinded - to prevent misuse in evaluating the results - he was unaware of the allocation of the groups. The study protocol was approved by the ethics committee gastroenterology and liver disease research center of Shahid Beheshti University of Medical Sciences (ID: IR.SBMU.RIGLD.REC.1401.042. Also, it was approved in the registry of the clinical trial center on 20/05/2023- https://irct.behdasht.gov.ir/trial/69397 (code: IRCT20220417054557N1).

### Participants

From March 2023 to August 2023, patients aged 18–50 with UC who were in remission based on laboratory criteria (stool calprotectin and normal CRP), endoscopy (zero Mayo) and clinical symptoms (absence of abdominal cramps, and bowel movements less than once a day) and suffered from IBS-liked symptoms based on ROME-IV criteria including recurrent abdominal pain on average at least 1 day/week in the last months associated with two or more of the changes in frequency of stool and/or change in the appearance of stool and/or increase related to defecation (26), participated in this study. Patients with pregnancy, other gastrointestinal diseases, the appearing of serotonin syndrome in patients receiving paroxetine and GFR < 30 in patients receiving gabapentin excluded from the study. Patients were also screened for the absence of psychological disorders and the absence of psychiatric medication by a psychiatrist. 100 patients referred to the Gastroenterology and Liver disease Clinic, Taleghani Hospital, Tehran, Iran were included in the study. Three people dropped out during the treatment because of some mild side effects, including drowsiness, nausea, and vomiting. Although they withdrew from the study due to unwillingness to continue treatment, the psychiatrist followed them up until complete recovery. Their symptoms improved without any therapeutic intervention. Ultimately, 97 patients remained until the end of the treatment period.

### Sample size

Based on the of Ford et al. (27), we assumed 25% different in outcome between two groups, with 80% power, 90% of confidence and 10% from drop out (in each group), we reached to 50 patients in each group. Supplementary Figure 1 show how to calculate the sample size.

### Randomization and intervention

After initial diagnosis of IBS-liked symptoms in UC patients who were in remission by a gastroenterologist, interventional process of study started. To ensure unbiased allocation, patients were randomly assigned to one of two treatment arms using a block randomization method. The block size was set at four, accommodating three patients per block in accordance with the order of enrollment. This randomization process was executed using the online block randomization software “Sealed Envelope,” and the randomized block lists were enclosed in sealed envelopes. Each day, a new envelope was opened by the gastroenterologist to determine the patient’s allocation.

**FIGURE 1:**
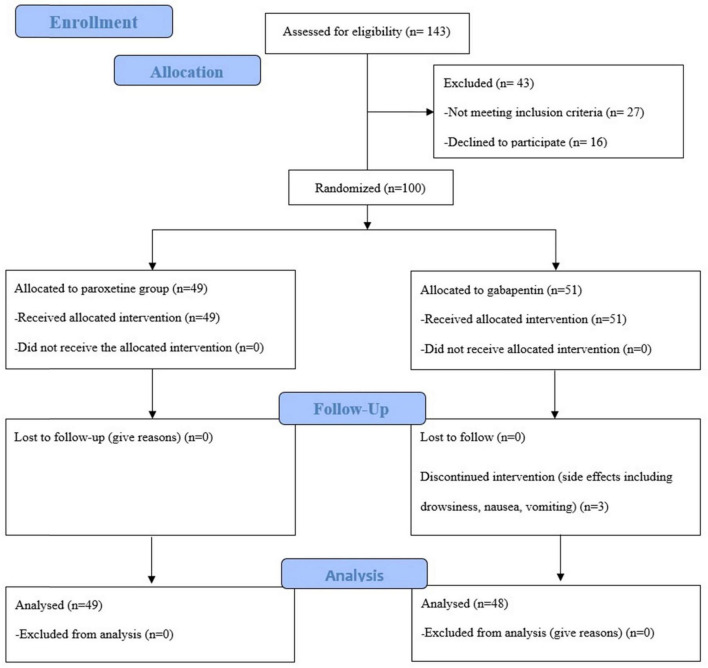
CONSORT flow diagram.

In the Paroxetine arm, patients were administered tablets containing 20 mg/day of paroxetine- -flexible during the 3 months to be taken postprandially. In the alternative treatment arm, patients received a daily postprandial dosage of 100 mg/day of Gabapentin in first month. After the end of the first month, according to the patient’s tolerance, the drug dose was increased to 300 mg per day. Patients were advised against the use of over-the-counter medications and not to change the dose of drugs arbitrarily. Forty-five days after the start of the treatment, the patients’ adherence to the medication was followed up by phone calls. After the 3-month treatment period concluded, the patients were re-visited and reevaluated. The CONSORT flow diagram (Figure 1) show the phases of this parallel randomized trial of two groups in details.

### Outcome assessment

Before the intervention, baseline characteristics including age, gender and IBS symptoms were recorded. The patients were assessed by four questionnaires:

*Short Form Health Survey questionnaire (SF-36):* this 36 items instrument evaluate Health-Related Quality of Life and measure eight scales including physical functioning, role physical, bodily pain, general health, vitality, social functioning, role emotional and mental health. The questionnaire employs different Likert scale formats, including two-way, 6-option Likert, 5-option Likert, and 3-option Likert scales, to capture responses effectively. The lowest score in this questionnaire is 0 and the highest score is 100, and a higher score indicates a better health status (28). The Cronbach’s alpha coefficients for each scale of SF-36 in the Persian version, range from 0.77 to 0.90 except for the vitality scale (α = 0.65) (29). In the present study the Persian language version was used.

*The Hospital Anxiety and Depression Scale (HADS):* This 14-question questionnaire consists of two subscales of anxiety (7 questions) and depression (7 questions) and is implemented in the population without psychiatric disorders. Each item is scored on a 4 -point Likert scale ranging from 0 to 3, with a score range of 0–21 for both subscales. Higher scores indicate a greater anxiety and depression state (30). Cronbach’s alpha coefficient has been found to be 0.78 for the HADS anxiety sub-scale and 0.86 for the HADS depression sub-scale in the Persian version (31). In the present study the Persian language version was used.

*IBS Severity Scale*: This scale gauges the severity of particular IBS symptoms and is capable of detecting changes over time. It utilizes a maximum score of 500, with scores under 75 indicating normal bowel function. Mild IBS falls within the 75–174 score range, moderate IBS within 175–299, and severe IBS within 300–500 (32). The total reliability of the questionnaire has been reported by using Cronbach’s alpha as 0.95, ranging from 0.65 to 0.90 in the Persian version which has been used in the present study (33).

The assessment was performed at baseline. After 3 months the intervention stope and patients reassessed. The primary outcome was a change in the severity of IBD symptoms and the secondary outcome was an improvement in the level of quality of life, anxiety, and depression. UC remaining in remission and not reactivating symptoms was assessed by clinical examination and according to Mayo.

### Bias

To avoid bias in this study, the person evaluating the outcomes and effects of the intervention did not know which patient received which drug, and the patients were referred to him with a code. Also, to avoid bias in data analysis and review of findings, the authors did not have access to information that could identify participants during or after data collection.

### Statistical analysis

All statistical analysis was performed using Statistical Packages for Social Sciences (SPSS) version 27 (IBM, Chicago, United States). Quantitative data are presented with mean with standard deviation or median with interquartile range, and qualitative data are presented with frequency and percentages. Comparison of quantitative data between the two groups were performed using Independent Sample *T*-test, and Wilcoxon-Rank Sum test and comparison of qualitative data were conducted using Pearson Chi-Square tests. Follow-up evaluations for quantitative data were performed using Wilcoxon Signed-ranked Test. Comparing the changes between the two groups was performed using Repeated Mixed ANOVA. Furthermore, Mediation analysis was performed using PROCESS Macro extension by Andrew F. Hayes. A *p* < 0.05 was considered significant.

## Results

### Demographics

A total of 97 patients met the inclusion criteria, with 29 (29.9%) experiencing constipation type, 44 (45.4%) exhibiting diarrhetic type, and 24 (24.7%) presenting with mixed form IBS-like symptoms. The mean age of the patients was 37.87 ± 4.50 years with 63.9% female predominance. The mean age in the paroxetine Group was 37.90 ± 4.36 with 59.2% female proportion and the mean age in the gabapentin group was 37.83 ± 4.68 which 68.8% were female. Throughout the study period, all patients remained in remission. No significant differences were observed between the two study groups in terms of age (*p* = 0.997) or sex (*p* = 0.399). A more detailed description of the demographic findings is presented in Table 1.

**TABLE 1:** Baseline characteristics of the participants in each treatment group.

Characteristics	Levels	Groups			*P*-value^1^
		**Total (*N* = 97)**	**Gabapentin (*N* = 48)**	**Paroxetine (*N* = 49)**	
Age		38.00 (34.00, 42.00)	38.00 (34.00, 42.00)	38.00 (33.50, 41.50)	0.997
Sex	Male	35 (36.1)	15 (31.3)	20 (40.8)	0.399
	Female	62 (63.9)	33 (68.8)	29 (59.2)	
IBS	IBS-C	29 (29.9)	15 (31.3)	14 (28.6)	0.932
	IBS-D	44 (45.4)	22 (45.8)	22 (44.9)	
	IBS-Mixed	24 (24.7)	11 (22.9)	13 (26.5)	

^1^Wilcoxon Rank-Sum Test, Pearson’s Chi Square Test. Quantitative data are presented as median with interquartile range. Qualitative data are presented as frequency and percentages. IBS, irritable bowel syndrome.

### Anxiety

The mean pre-intervention anxiety score in the paroxetine group was 15.75 ± 1.94, and in the gabapentin group was 14.84 ± 2.43. The follow-up evaluation within 3 months revealed a mean score of 15.50 ± 1.98, and 13.24 ± 2.29 in the paroxetine and gabapentin groups, respectively. Pre-intervention anxiety scores were not significantly different between the two groups (*p* = 0.080); however, post-intervention evaluation revealed significantly lower anxiety levels among patients in the paroxetine group. Paired evaluation in each treatment group revealed a significant reduction in the paroxetine group (*p* < 0.001) while changes in the gabapentin group were not significant (*p* = 0.071). Moreover, further investigation of anxiety scores based on sex revealed a significant reduction in both groups (*p* < 0.001), with male participants having significantly lower anxiety scores (*p* = 0.005). The anxiety score is presented in Table 2.

**TABLE 2:** Patients’ findings before and after intervention in each treatment group.

Characteristics	Groups	Time		*P*-value^1^	*P*-value^2^
		**Pre-intervention**	**Post-intervention**		
Quality of life	Gabapentin	424.00 (383.67, 446.17)	428.17 (382.17, 443.84)	0.479	0.010
	Paroxetine	417.67 (377.83, 457.67)	472.00 (442.67, 490.00)	<0.001	
	*P*-value^3^	0.722	<0.001		
Anxiety	Gabapentin	16.00 (14.00, 17.00)	16.00 (14.00, 17.00)	0.071	<0.001
	Paroxetine	15.00 (13.00, 17.00)	14.00 (12.00, 15.00)	<0.001	
	*P*-value	0.080	<0.001		
Depression	Gabapentin	14.50 (13.00, 16.00)	14.00 (13.00, 15.00)	0.101	0.424
	Paroxetine	15.00 (13.00, 16.00)	13.00 (12.00, 14.00)	<0.001	
	*P*-value	0.196	0.005		
Severity	Gabapentin	111.00 (78.00, 163.50)	109.00 (80.00, 152.50)	0.028	0.338
	Paroxetine	107.00 (71.00, 167.00)	86.00 (64.00, 112.00)	<0.001	
	*P*-value	0.691	0.045		

^1^Wilcoxon Signed-Rank Test.

^2^Repeated Mixed ANOVA.

^3^Independent Sample *T*-test, Wilcoxon Rank-Sum Test. Quantitative data are provided as median with interquartile range.

### Depression

The mean depression score in the paroxetine group before intervention was 14.92 ± 2.19, and in the gabapentin group was 14.38 ± 2.22. Post-intervention assessments revealed a mean score of 12.88 ± 2.10, for the paroxetine group and 14.08 ± 2.05 for the gabapentin groups. Pre-intervention depression scores were not significantly different between the two groups (*p* = 0.196); however, post-intervention evaluation revealed significantly lower depression scores among patients in the paroxetine group. Depression score significantly reduced in the paroxetine group (*p* < 0.001) while changes in the gabapentin group were not significant (*p* = 0.101). Comparison of the depression score based on sex revealed a significant reduction in both groups (*p* < 0.001), with no significant difference between the two groups (*p* = 0.080). Table 2 presents data regarding the depression score.

### IBS severity

Prior to intervention, the IBS severity score in the paroxetine group was 139.71 ± 109.90, and in the gabapentin group, it was 149.90 ± 119.39. Follow-up evaluation revealed a mean score of 113.20 ± 88.57 in the paroxetine group and 145.06 ± 112.118 in the gabapentin group. Pre-intervention severity scores did not differ significantly between the two groups (*p* = 0.691); however, post-intervention evaluation revealed significantly lower severity scores among patients in the paroxetine group (*p* = 0.045). Paired evaluation in each treatment group showed a significant reduction in both gabapentin (*p* = 0.028), and paroxetine (*p* < 0.001) groups, while the paroxetine group demonstrated significantly more pronounced reductions (*p* < 0.001). Comparison of the severity score based on sex revealed significant reduction in both groups (*p* < 0.001), with no significant difference between the two groups (*p* = 0.055). Table 2 presents data regarding the IBS severity score.

### Quality of life

Before the intervention, the quality-of-life score in the paroxetine group was 52.04 ± 6.52, and in the gabapentin group was 51.62 ± 7.39. Follow-up evaluation within 3 months revealed mean scores of 71.07 ± 6.51, and 52.73 ± 6.94 in the paroxetine and gabapentin groups, respectively. Pre-intervention quality-of-life scores were not significantly different between the two groups (*p* = 0.934); however, post-intervention evaluation revealed significantly higher quality-of-life scores among patients in the paroxetine group (*p* < 0.001). Paired evaluation within each treatment group revealed significant increments in the paroxetine group (*p* < 0.001), while changes in the gabapentin group were not significant (*p* = 0.06). Table 2 presents data regarding the quality-of-life score.

**FIGURE 2:**
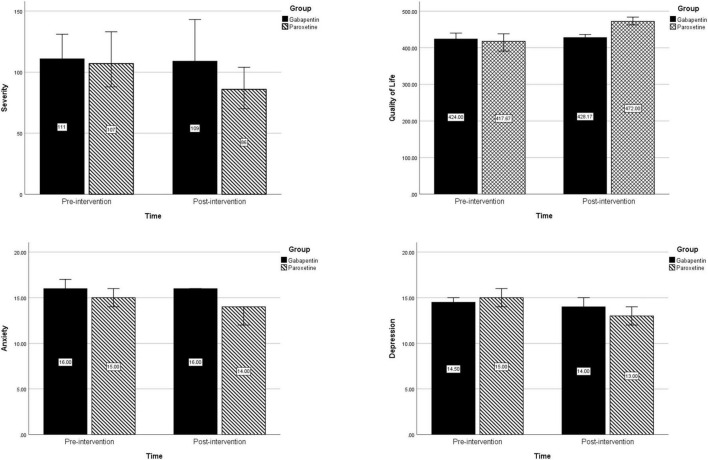
Changes in severity, quality of life, depression, and anxiety before and after intervention in each treatment group.

An overview of changes in severity, quality of life, depression, and anxiety in each treatment group is presented in Figure 2.

### Mediation analysis

**FIGURE 3:**
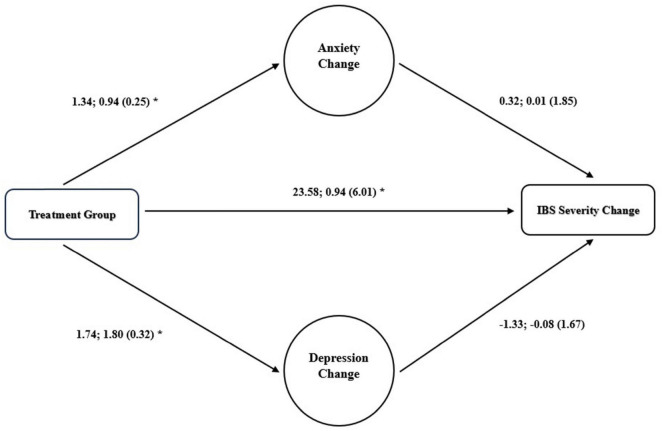
Mediation role of changes in anxiety for improvement in IBS severity. Unstandardized regression correlations; standardized correlations (standard error) are presented, *p < 0.05.

A mediation analysis evaluating the role of anxiety and depression alongside pharmacologic treatment in improving severity scores revealed no significant mediation role for anxiety (*p* = 0.859) or depression (*p* = 0.424). Figure 3 provide an illustration of the mediation analysis.

## Discussion

In this study, we conducted a randomized single-blind clinical trial involving a total of 97 patients, evenly divided into two treatment arms: 49 patients received treatment with paroxetine, while 48 patients received treatment with gabapentin. We assessed several key outcomes, including the levels of anxiety and depression, the quality of life.

In this study, all evaluated patients experienced improvements in the severity of IBS symptoms, a reduction in anxiety and depression, and an enhanced quality of life in both treatment groups after 3 months of intervention compared to their pre-intervention status. Significantly, the group treated with paroxetine exhibited marked improvements in all areas, while the gabapentin group showed significant improvement only in the severity of IBS symptoms. Both groups remained in remission. A comparative analysis between the two treatment groups revealed that paroxetine outperformed gabapentin in terms of alleviating symptom severity, reducing anxiety and depression, and enhancing quality of life. Notably, some patients using gabapentin reported side effects, including drowsiness, nausea, and vomiting. As a result, three patients (out of the initial 100 patients) were excluded from the study due to these side effects, a phenomenon consistent with findings in prior studies (34–36) that have also reported dizziness and drowsiness associated with gabapentin use.

Based on our findings, paroxetine offers some advantages, notably in enhancing the quality of life and alleviating both digestive and psychological functional symptoms in UC patients in remission with IBS-liked symptoms. These results align with the study conducted by Stasi et al. (37), who observed significant improvements in psychiatric and gastrointestinal symptoms when IBS patients with moderate to severe symptoms and psychiatric disorders were treated with low-dose paroxetine (5 mg/day) in a longitudinal evaluation. Lu et al. (38) also reported similar positive effects in IBS patients after 7 weeks of daily paroxetine administration (20–40 mg), with notable improvements in gastrointestinal symptoms, particularly diarrhea, followed by a reduction in psychological symptoms such as anxiety and somatization While our research found no recent clinical trials investigating paroxetine’s effects on IBS patients, older studies, revealed varying outcomes regarding pain symptoms. In a study by Masnad et al. (39) a 12-week treatment with paroxetine (12.5–50 mg) resulted in enhanced global effect and severity-improvement ratings, although it did not lead to a reduction in pain scores. The authors emphasized that despite paroxetine’s limited impact on abdominal pain symptoms, it offers broad and potential benefits to individuals with IBS. A study conducted by Tabas et al. (40) found that a 12-week treatment with paroxetine (10 mg daily) did not significantly alleviate symptoms related to abdominal pain, bloating, or social functioning compared to a placebo. However, they noted a notable increase in the overall sense of well-being. Additionally, Creed et al. (41) further supported the effectiveness of paroxetine in IBS management. A common feature in all these studies was the patients’ good tolerance of paroxetine, which aligns with our findings.

Serotonin, a neurotransmitter present in both the central nervous system and the intestinal nervous system, plays a vital role in regulating intestinal motility. In IBS, disruptions in serotonin synthesis and secretion are linked to the onset of symptoms (42). Reduced intestinal serotonin production has been associated with a weakened intestinal lining, leading to cramping or constipation, while elevated serotonin levels in the gut can also contribute to symptoms (43). Selective serotonin reuptake inhibitors (SSRIs), the most commonly used medications for anxiety and depression, work by increasing synaptic serotonin levels. By inhibiting the reuptake of serotonin from the synaptic space, SSRIs elevate serotonin levels in the brain (44, 45). Studies in rats have shown that paroxetine, an SSRI, can reduce stool production and delay gastrointestinal motility (46). It is suggested that paroxetine’s impact on mucosal serotonin signaling mechanisms contributes to symptom improvement in IBS (47). Moreover, there is evidence supporting the anti-inflammatory properties of SSRIs (23). Previous research has indicated that paroxetine can inhibit the expression of various inflammatory factors at the mRNA level and reduce inflammation-related pathways (48). Since inflammation is known to be involved in the pathogenesis of IBS (49), it is plausible that paroxetine’s ability to reduce inflammation has led to symptom improvement.

In our study, we also investigated the effectiveness of gabapentin, a drug with known antidepressant (50), anti-inflammatory (51) and anti-neural pain properties, often used in IBS patients (36). This led us to hypothesize that gabapentin might positively impact gastrointestinal functional symptoms in UC patients in remission. Our results revealed that gabapentin did lead to a significant improvement in the severity of IBS symptoms, suggesting its potential utility in this context. However, it’s worth noting that the improvement in symptom severity was more pronounced with paroxetine compared to gabapentin. The superior effect of paroxetine in managing both gastrointestinal and psychological symptoms can be attributed to its multi-faceted impact on the gut-brain axis (52). By selectively inhibiting the reuptake of serotonin, paroxetine enhances serotonergic signaling in both the brain and the gut, which may explain its broad therapeutic effects on both psychiatric and gastrointestinal symptoms in IBS patients (53). Moreover, SSRIs like paroxetine are known to modulate the activity of serotonin receptors in the enteric nervous system, which helps reduce visceral hypersensitivity—a core feature of IBS (46).

Also, gabapentin increases the level of serotonin in the whole blood Lee et al. demonstrated that gabapentin (300–600 mg daily) reduced rectal sensory thresholds, resulting in decreased rectal sensitivity to distension and increased rectal compliance in IBS patients (35). Additionally, Zhang et al. reported that gabapentin had a pain and anxiety-inhibiting effect in rats with IBS (54). While our study showed that gabapentin had some positive impact on anxiety, depression, and quality of life, these improvements were not statistically significant. This finding aligns with a review study by Berlin et al., who suggested that while gabapentin might be useful for some anxiety disorders, clear evidence for its efficacy in addressing anxiety and depression remains inconclusive (55). The lesser efficacy of gabapentin than paroxetine could be because gabapentin, although it possesses some serotonergic and anti-neural properties, primarily acts through the inhibition of calcium channels, thereby reducing neuronal excitability. While this mechanism can alleviate neuropathic pain and modulate sensory processing, its impact on the serotonergic system is less direct than that of SSRIs (56, 57).

Another noteworthy finding from our study is that both the gabapentin and paroxetine groups of patients remained in remission, with no UC relapse observed. This outcome may partly be due to the known anti-inflammatory properties of these drugs (48, 51). Paroxetine, for example, has been shown to reduce intestinal inflammation by effectively managing the microbial balance within the digestive system (48). Similarly, gabapentin’s role in preventing inflammation is attributed to its regulation of mast cell signaling and its capacity to reduce the activation of inflammatory genes associated with inflammatory bowel diseases (58). This anti-inflammatory action may explain the sustained remission observed in both treatment groups in our study.

The present study boasts several strengths, including a 3-month follow-up period for patients, the focus on individuals with no prior history of psychiatric drug use, and the examination of the effects of two distinct drugs, paroxetine and gabapentin, on UC patients in remission with IBS-liked symptoms. However, we acknowledge certain limitations our study was single-centered, and we did not conduct long-term follow-ups to investigate the recurrence of symptoms following the discontinuation of these drugs. To address this gap, future research should encompass multicenter studies and implement extended follow-up periods to assess symptom recurrence rates after treatment cessation. Furthermore, we use low Gabapentin dosage which is lower than therapeutic dosage. Considering that this low dose was well tolerated in the patients of this study, we suggest that future studies investigate standard therapeutic doses in this group of patients. Lack of control on several confounding factors like type of IBS-like syndromes is another limitation of our study that should be investigated clearly in the future studies. Also, Limitations of our study include the lack of specific examination and documentation of side effects associated with the medications used in the trial. While adverse events were monitored and managed throughout the study, including the exclusion of three patients due to gabapentin-related side effects, we did not systematically collect data on all potential side effects. This omission limits our ability to comprehensively assess the safety profiles of gabapentin and paroxetine in this context. Future studies should incorporate more robust methods for monitoring and reporting side effects, including standardized assessments and documentation procedures, to provide a more thorough understanding of the risks associated with these medications in patients with UC and irritable bowel syndrome-like symptoms.

## Conclusion

In conclusion, paroxetine exhibited significant improvement effects in gastrointestinal functional symptoms, anxiety, depression, and quality of life in UC patients in remission. On the other hand, while gabapentin led to a significant improvement in IBS symptoms, it did not yield significant improvements in anxiety, depression, and quality of life. It’s worth noting that the rate of improvement in gastrointestinal functional symptoms, anxiety, depression, and quality of life was significantly superior with paroxetine compared to gabapentin.

## Data Availability

The raw data supporting the conclusions of this article will be made available by the authors, without undue reservation.
