# Supplementary material for: Unveiling the efficacy of paroxetine and gabapentin in ulcerative colitis patients in remission with co-existing IBS-like symptoms: a single-blinded randomized clinical trial
Source: Front Med (Lausanne). 2024 Nov 20;11:1468885. doi: 10.3389/fmed.2024.1468885 (PMC11614664; doi:10.3389/fmed.2024.1468885)
Supplement: Supplementary file 1 [file Data_Sheet_1.docx]

**Supplementary files**

**Tables**

[Table 2 Quality of Life subscales pre- and post-intervention 2](#_Toc180922716)

[Table 3 Quality of Life subgroups pre- and post-intervention 3](#_Toc180922717)

**Figures**

[Figure 1 recommended sample size for this specific research (source: https://select-statistics.co.uk/ , 2022) 4](#_Toc180909016)

[Figure 2 Mediation role of changes in anxiety for improvement in IBS Severity; *: p<0.05 5](#_Toc180909017)

[Figure 3 Mediation role of changes in depression for improvement in IBS Severity; *: p<0.05 6](#_Toc180909018)

**Table 2** Quality of Life subscales pre- and post-intervention

| **Levels** | **Study Groups** |  | **Assessment** | | **p-value** |
| --- | --- | --- | --- | --- | --- |
|  |  |  | Pre-intervention | Post-intervention |  |
| **Physical Health** | Total |  | 211.36± 37.19 | 223.29± 39.15 | <0.001 |
|  | Gabapentin |  | 203.75± 38.50 | 206.95± 36.54 |  |
|  | Paroxetine |  | 218.82± 34.64 | 239.30± 35.06 |  |
|  |  |  |  |  |  |
| **Psychological Health** | Total |  | 202.96± 46.44 | 217.88± 43.37 | <0.001 |
|  | Gabapentin |  | 208.55± 49.37 | 208.09± 40.88 |  |
|  | Paroxetine |  | 197.50± 43.19 | 227.46± 43.99 |  |

**Table 3** Quality of Life subgroups pre- and post-intervention

| **Levels** | **Study Groups** |  | **Assessment** | | **p-value** |
| --- | --- | --- | --- | --- | --- |
|  |  |  | Pre-intervention | Post-intervention |  |
| **Physical functioning** | Gabapentin |  | 51.77±13.10 | 53.02± 12.23 | 0.059 |
|  | Paroxetine |  | 52.04± 13.46 | 56.84± 12.52 | <0.001 |
|  |  |  |  |  |  |
| **Role limitation due to physical health** | Gabapentin |  | 47.92± 24.09 | 48.96± 23.60 | 0.670 |
|  | Paroxetine |  | 52.04± 23.87 | 65.31± 22.71 | <0.001 |
|  |  |  |  |  |  |
| **Role limitation due to emotional problems** | Gabapentin |  | 56.25± 28.48 | 52.77± 23.65 | 0.037 |
|  | Paroxetine |  | 44.89± 25.95 | 59.18± 29.08 | <0.001 |
|  |  |  |  |  |  |
| **Energy Fatigue** | Gabapentin |  | 49.58± 16.56 | 50.83± 15.37 | 0.070 |
|  | Paroxetine |  | 49.39± 16.44 | 53.57± 15.51 | <0.001 |
|  |  |  |  |  |  |
| **Emotional Well-being** | Gabapentin |  | 49.33± 14.99 | 50.04± 14.91 | 0.352 |
|  | Paroxetine |  | 53.47± 13.28 | 57.59± 12.61 | <0.001 |
|  |  |  |  |  |  |
| **Social Functioning** | Gabapentin |  | 53.39± 23.59 | 54.44± 20.69 | 0.012 |
|  | Paroxetine |  | 49.74± 26.69 | 57.12± 23.38 | <0.001 |
|  |  |  |  |  |  |
| **Pain** | Gabapentin |  | 51.46± 23.67 | 52.06± 21.74 | 0.357 |
|  | Paroxetine |  | 58.32± 19.68 | 60.06± 18.46 | 0.016 |
|  |  |  |  |  |  |
| **General Health** | Gabapentin |  | 52.60± 17.65 | 52.92± 16.49 | 0.590 |
|  | Paroxetine |  | 56.43± 16.83 | 57.10± 16.11 | 0.297 |


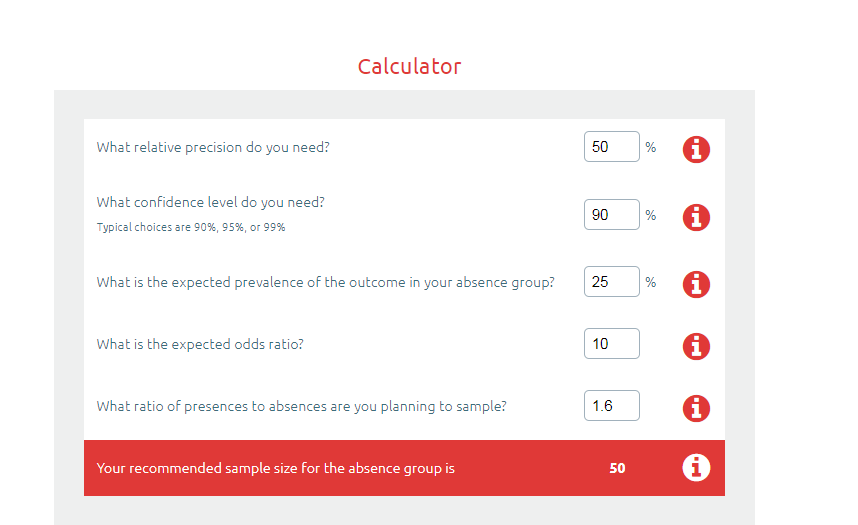


**Figure 1** recommended sample size for this specific research (source: https://select-statistics.co.uk/ , 2022)

HADS Anxiety difference

-1.34 (-0.94) *

-21.22 (-0.85) *

Treatment Group

IBS-SSS difference

0.33 (0.01)

**Figure 2** Mediation role of changes in anxiety for improvement in IBS Severity; *: p<0.05

-1.34 (-0.08)

-1.74 (-1.70) *

**Figure 3** Mediation role of changes in depression for improvement in IBS Severity; *: p<0.05

Treatment Group

IBS-SSS difference

HADS Depression difference

-24.02 (-0.96) *
